# Post-Oberlin procedure cortical neuroplasticity in traumatic injury of the upper brachial plexus

**DOI:** 10.1590/0100-3984.2015.0082

**Published:** 2016

**Authors:** Ana Caroline Siquara de Sousa, José Fernando Guedes-Corrêa

**Affiliations:** 1 Hospital Universitário Gaffrée e Guinle - Universidade Federal do Estado do Rio de Janeiro (Unirio), Rio de Janeiro, RJ, Brazil.

*Dear Editor*,

A 27-year-old left-handed male injured his left arm in a motorcycle accident. The clinical examination showed a lack of movement in the left forearm and shoulder, with normal movement of the left hand. Magnetic resonance imaging (MRI) showed avulsion of left upper nerve roots (C5 and C6) of the brachial plexus, caused by traumatic lesion. He underwent neurotization by the Oberlin procedure and transfer of the accessory nerve to the suprascapular nerve three months after the accident^(1)^. The first signs of re-innervation of the biceps muscle appeared two months after the surgical procedure. The patient later showed significant signs of recovery.

We selected this patient to undergo functional MRI (fMRI). For the fMRI acquisition, we also selected one healthy control volunteer who was matched to the patient for age, gender, and handedness. Both subjects underwent MRI in order to compose a structural sequence with anatomical images. In the functional sequence, we employed the following acquisition parameters: repetition time = 2000 ms; echo time = 30 ms; flip angle = 90°; matrix = 64 × 64; field of view = 240 mm; voxel resolution = 3.75 × 3.75 × 5 mm; slice thickness = 5 mm; sagittal plane, 22 planes.

**Figure 1:**
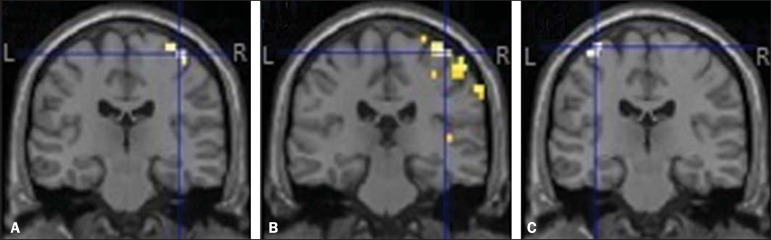
Cortical activation during motor tasks in the patient. Only the cortex is represented, and the peak of activation in the coordinates were as follows: **A:** left hand, x = 38 mm, y = -22 mm, z = 65 mm; **B:** left elbow, x = 42 mm, y = -26 mm, z = 65 mm; **C:** right elbow, x = -33 mm, y = -18 mm, z = 70 mm. (R, right hemisphere; L, left hemisphere).

The motor tasks consisted of elbow flexion and hand grasping, in a "block" design: hand grasping of the dominant injured limb (left upper limb) and elbow flexion of the injured and healthy limbs. All motor tasks were alternated with a rest period (there were 100 dynamics per block, and there were 10 rest dynamics for each set of 10 task dynamics). Ten blocks of each state (resting and limb movement) were used. The patient and the healthy volunteer performed the same tasks. The MRI of the patient showed no anatomical alterations. After family-wise error correction at a value of *p* < 0.05, all fMRI scans acquired during the motor tasks showed main activation of the contralateral hemisphere in the areas that correspond to the primary motor cortex (Figure 1), as follows: forearm and hand for hand grasping of the left upper limb; arm, forearm, hand, and face for left elbow flexion; arm and forearm for flexion of the right upper limb. The MRI of the healthy volunteer also showed no anatomical alterations.

In the case reported here, fMRI was effective in identifying the cortical activations. The comparison between the patient and the healthy volunteer showed that the areas of cortical activation were quite similar, as were the activation peaks. The detectable reactivation of the cortical area in the patient during flexion of the injured elbow corresponded to the arm area in the motor homunculus of the volunteer^(2-8)^. The cortical activations in this case were similar to those reported in previous studies that applied extraplexus nerve transfer techniques, the areas of activation mainly being located in the contralateral cortex^(2-8)^.

This study has some limitations. We presented the patient data only in comparison with those of a single control participant, rather than with a group of control, and both data sets were acquired at only one time point. In addition, the patient did not undergo a pre-operative fMRI scan.
